# Nitrate Reductase Inhibition Induces Lipid Enhancement of Dunaliella Tertiolecta for Biodiesel Production

**DOI:** 10.1155/2018/6834725

**Published:** 2018-12-02

**Authors:** Redouane Benhima, Hicham El Arroussi, Issam M. Kadmiri, Najib El Mernissi, Imane Wahby, Iman Bennis, AbdelAziz Smouni, Najib Bendaou

**Affiliations:** ^1^Green Biotechnology Centre, Moroccan Foundation for Advanced Science Innovation and Research, Morocco; ^2^Laboratoire de Physiologie et Biotechnologie Végétale, Faculté des Sciences, Mohammed V University, Morocco

## Abstract

Nitrogen stress increases lipids content in microalgae, the main feedstock for algal biodiesel. Sodium tungstate was used in this study to implement nitrogen stress by inhibiting nitrate reductase (NR) in* Dunaliella tertiolecta*. The reduction of NR activity was accompanied by reduction of chlorophyll and accumulation of lipids. One-stage and two-stage culture strategies were compared. One-stage culture raised total lipids from 18% (control) to 39% (w: w); however, two-stage culture raised lipids to 50% in which neutral lipids were enhanced 2.14 times. To assess the quality of biodiesel produced, fatty acid methyl esters (FAME) composition was studied. It showed a slight variation of unsaturation. In addition, some physical proprieties of biodiesel were estimated and showed that higher heating values were improved by tungstate treatment. In this study, we tried to shed light on some biological impact of NR inhibition in microalgae cells using sodium tungstate which could be exploited in the improvement of biodiesel production.

## 1. Introduction

The increase of energy consumption during the previous century, the depletion of fossil fuels and the accumulation of greenhouse gases led to the emergence of renewable energies as promising alternative.

Renewable energy provided an estimated 18.2% of global final energy consumption in 2016 [1]. Bioenergy is the largest renewable contributor to global final energy demand, providing nearly 13% of the total. This production is constantly increasing. Indeed, biofuels production for transport increased 2.5% in 2017 [1].

Biodiesel is a bioenergy produced from a variety of sources. Microalgae are one of the most promising sources of biomass for biodiesel production thanks to their high lipid content [1]. However, biodiesel from microalgae is not yet in the market due to its noneconomical viability at large scale. Some locks should be lifted starting by the improvement of growth rate and lipid content which are the key parameters of lipid productivity.

*Dunaliella tertiolecta*, marine green flagellate microalgae, has a potential for the biodiesel production [2–4]; however, it needs some improvements of its lipid content [5]. Previous studies have demonstrated that the quantity of lipids varies according to the change of growth conditions such as nutrients concentrations, light intensity, or temperature [6, 7]. This enables microalgae to accumulate a significant amount of neutral lipids under stress conditions. Nutritional stress like nitrogen deprivation, phosphorus starvation, and iron supplementation can enhance the lipid content in many microalgae species [8–11].

NR inhibition as a tool of nitrogen starvation in microalgae has not been widely investigated. In eukaryotic microalgae, nitrate reductase (NR) is a molybdoenzyme reducing nitrate to nitrite:

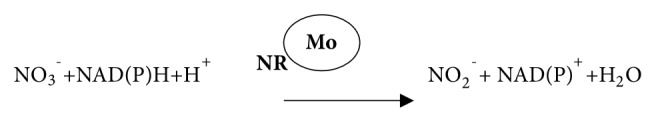
(1)This reduction constitutes the first step in the assimilation of nitrate before obtaining the ammonium which is incorporated into Glutamate through GS/GOGAT pathway in chloroplast. The inhibition of NR can be caused by tungsten [12].

Nitrogen stress, by NR inhibition or other technique, led to accumulation of lipid in microalgae cells but at the same time it severely reduces growth rate. In the present study, we attempted to overcome this problem using an easy and low-cost two-stage culturing strategy. Furthermore, we investigated the impact of nitrogen stress on* Dunaliella tertiolecta *cells, lipid compositions, and most important proprieties of produced biodiesel.

## 2. Materiel and Methods

### 2.1. Microalgae Strains and Growth Conditions

*Dunaliella tertiolecta *was isolated from Oualidia lagoon in Morocco and maintained in MAScIR's Microalgae Collection (Moroccan Foundation for Advanced Science, Innovation and Reseach). Strain was cultivated in sterilized seawater which was previously filtered by using 0.45 um cellulose nitrate filter was and enriched by F/2 medium [13] and cultures were set up in 250 ml Erlenmeyer flasks containing 150 mL of volume culture with initial OD 0.1 at 24°C ± 2°C under continuous illumination at 150 *μ*mol m^−2^s-^1^ and the agitation of culture was 125 rpm [5].

### 2.2. Tungsten Treatment

In one stage culture strategy, cells were treated by sodium tungstate at the beginning of culture. In two-stage culture strategy, cells were conducted in Guillard F/2 medium until the 7th day when 10 mM of sodium tungstate was added. The choice of the 7th day was based on a previous experience (data not shown). Controls were maintained in Guillard F/2 until the 15th day. The experiment was carried on during 15 days in triplicate.

### 2.3. Growth Measurement

Microalgae growth was monitored during the days of cultures by measuring optical density at 680 nm (Ultraspec 3100 pro). After harvesting biomass by centrifugation at 4500 rpm for 5 minutes and drying it by lyophilization (at Christ Alpha 2-4 LSC Freeze Dryer) for 16 h at -80°C, dried biomass was weighted with digital analytical balance Sartorius [2].

### 2.4. Total Lipid Extraction

Total lipid extraction was performed according to our protocol [5] which was a modified protocol of [14]. Lyophilized microalgal biomass with 2% of BHT (Butylated hydroxytoluene) and chloroform was treated by ultrasonic (Branson Sonifier 450): 40 KHz for 15 min at room temperature. Lipids were extracted using a solvent mixture: water/chloroform/methanol (0.8/2/1). The extraction mixture was centrifuged at 5000 rpm for 5 min and the lower phase was recovered. Extraction was made twice. Cleaning was made by sodium chloride solution (0.9% w/v) using separating funnel. Finally, chloroform was evaporated by nitrogen gas flow and total lipids were weighed [1].

### 2.5. FAMEs Determination

The fatty acid methyl esters (FAME) profile of* D. terliolecta* was determined after basic transesterification according to our protocol [8, 15]. The reaction was catalyzed by 2% NaOH (w:w) in methanol 1:20 at 80°C and atmospheric pressure during 6 hours. FAME profile was characterized by gas chromatography (GC) (Agilent 7890A Series GC) coupled to mass spectrometry (MS) equipped with multimode injector and 5-ms column (30 m x 250 um x 0,25 um) and electron impact ionization.

Two *µ*L of FAME solubilized in chloroform was injected into column by splitless mode using helium as carrier gas at 1.5 mL/min. The ion source and quadruple temperatures were 230°C and 150°C, respectively. The oven temperature program was started at 70°C and maintained 1 min, increased at 20°C/min until 120°C, was then, held one minute before to be increased until 200°C by 30°C/min and held one minute then, increased at 250°C at 10°C/min and held one minute, then increased until 270°C at 5°C/min, and finally kept constant for 5 min. FAME composition was calculated as percentage of the total FAMEs presents in the sample, determined from the peak areas. Detection was done using full scan mode between 35 to 600 m/z and with gain factor 5 and the identification was performed using NIST 2011 MS Library and confirmed by known standards Supelco® 37 Component FAME Mix (47885-USigma Aldrich) [2].

### 2.6. Determination of Physical Proprieties of Biodiesel

Physical properties of each FAME including cetane number, Kinematic viscosity, Density and Higher heating value were calculated from empirical equations [16]:

(2) Cetane  number=−7.8+0.302 . Mi−20 . Nln⁡Kinematic  viscosity=−12.503+2.496 . ln⁡Mi−0.178 . N)Density=0.8463+4.9Mi+0.0118 . NHigher  heating  value=46.19−1794Mi+0.21 . N

where *Mi* and *N* are the molecular weight of the *i*th FAME and the number of double bounds, respectively.

Then, each physical propriety of biodiesel was calculated using the following expression:

(3) $$\begin{array}{c} fb=\overset{n}{\underset{i=0}{\sum }}Pi\;.\;fi \end{array}$$

where *fb* is the physical propriety of biodiesel and *fi* is that the physical propriety of the *i*th FAME and Pi is the percentage of the *i*th FAME.

### 2.7. Neutral Lipids Analysis

#### 2.7.1. Microscopy

At stationary phase, 10 mL of the culture solution was collected and centrifuged at 5000 rpm for 5 min. Algal pellets were mixed with 3 mL of filtered seawater. Nile red (9-diethylamino-5H-benzo [*α*] phenoxa-phenoxazine-5-one) at analytical grade purchased from Sigma-Aldrich was used to stain neutral lipids. After addition of 10 *µ*L of Nile red solution (1 mg/mL in acetone), the mixture was incubated at 37°C for 1min, and stained in the dark for 10 min, then, lipid droplets were visualized under fluorescent microscope (Leica, DM 2500). 100 x objective lens was used to visualize the fluorescent yellow-gold lipid in microalgal cells [17].

#### 2.7.2. Spectrofluorimetry

Neutral lipids were quantified by spectrofluorimety (Agilent cary eclipse) according to our protocol [2]. Nile red (1 mg/mL of acetone) was added to 5 mL of triolein and solution mixed and incubated at room temperature for 10 min. Standard curve was drawn using different concentrations (1, 2, 5, 10, and 20 mg/mL) of triolein.* D. tertiolecta* culture (1.5 10^6^ cells) was centrifuged at 2000 rpm for 15 min and pellet was solubilized in 3 mL filtered sterile sea water. 3 mL of Nile red working solution was added to sample simultaneously with DMSO (10%) and mixture was incubated at room temperature for 10 min. Excitation and emission wavelength were 480 and 570 nm, respectively.

### 2.8. *In Situ* Determination of NR Activity

*In situ *method for measuring nitrate reductase (NR) activity in 15 days old* D. tertiolecta *cultures was used according to [18] with slight modification. 1.5 mL of microalgae culture from different treatments (5 - 30 10^6^ cell.ml^−1^) was made in NO_2_^−^ free medium and 4 mL of reaction mixture (0.1 M phosphate buffer, 0.5 mM EDTA, 5% (v/v) 1-propanol, 30 mM KNO_3_, 1 M NaCl) was added. All reaction tubes were separately bubbled with N_2_ prior adding cells. The tubes were quickly sealed with aluminum foil and incubated in the dark at 30°C under shaking (50 rpm) for 30 minutes. The reaction was stopped by filtering the assay mixture and filtered solution was used for nitrite determination. Nitrite was measured in the filtered solution by colorimetric method [19]. NR activity was expressed as *µ*mol NO_2_^−^ .10^6^ cells^−1^.h^−1^.

NR inhibition assay was performed using different sodium tungstate concentrations (0, 0.5, 1, 5, 7.5, 10, and 15 mM). Inhibitory concentration was compared with denaturing treatment by heating at 94°C for 10 min.

### 2.9. Chlorophyll Analysis

Effect of nitrogen depletion induced by tungstate addition on chlorophyll was studied. 20 mg of dried biomass were ground with 1 ml of 80% acetone and centrifuged during 1 min at 10,000 rpm. Acetone fraction was analyzed by spectrophotometry at two wavelengths, 645 and 663 nm. The Chlorophyll content (a and b) was determined using the following equations:* Chl*  *a* = 0.999_A663_–0.0989_A645_ and* Chl*  *b* = –0.328_A663_ + 1.77A_645_ [20].

## 3. Results

### 3.1. Effect of Tungstate on Microalgae Growth

**Figure 1:**
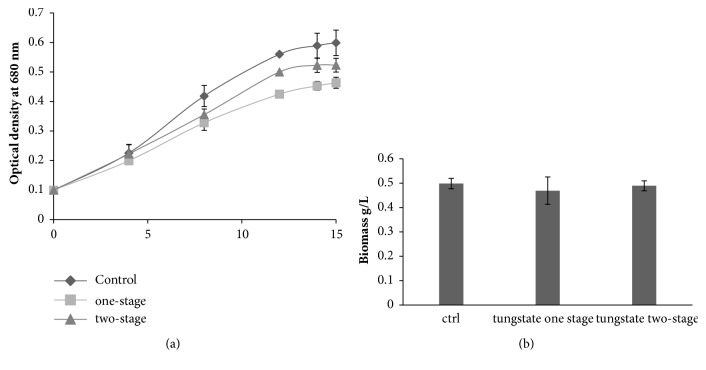
Effect of tungstate on growth of* D. tertiolecta*. (a) Monitoring of OD  _680 nm_ during 14 days. (b) Biomass accumulated at 15th days of culture (g/L). All cultures were cultivated in Guillard F/2 medium. Sodium tungstate was added to the cultures at the beginning of culture “one-stage treatment,” or at 7h day “Two-stage treatment.”

The growth of* D. tertiolecta* was compared in one-stage culture, two-stage culture and controls (Figure 1(a)). The maximal optical density (OD_max_) obtained at the 15th day in one-stage culture strategy was less than OD_max_ obtained in two-stage strategy culture: 0.46 ± 0.025 vs 0.5 ± 0.028 which is slightly less than control 0.6 ± 0.019 OD_max_. Average of specific growth rate was also calculated (*µ*_A_ = sloop of ln⁡(OD/OD_0_) vs time), and the result followed the same tendency with 0.126, 0.141 and 0.151 day^−1^, respectively. Biomass accumulated at the end of culture exhibited a decrease by 30% in comparison with the control which accumulates 0.46 g/L dry weight biomass, while two-stage treatment reduction was only 13% (Figure 1(b)).

### 3.2. Inhibition of Nitrate Reductase (NR) by Sodium Tungstate

**Figure 2:**
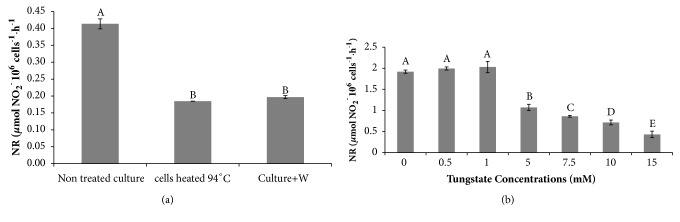
Impact of tungstate on NR activity (a) NR activity measurement in* D. tertiolecta* cells under different conditions: nontreated cells, heated cells (94°C), and cells treated with sodium tungstate after 15 days of culture at F/2. (b) Effect of concentration of sodium tungstate on NR activity in* D. tertiolecta* cells. Tested concentrations: 0, 0.5, 1. 5, 7.5, 10, and 15 mM. Statistical analysis was made using least significant difference (LSD) in SPSS.

Addition of tungsten which is the antagonist of molybdenum to the culture medium inhibits NR and subsequently inhibits the growth of microalgae by nitrogen depletion [15, 21, 22]. NR activity under several concentrations of tungstate was measured and results are shown in the Figure 2. Results show that NR activity at normal conditions was around 3 *µ*mol of NO_2_^−^ 10^6^ cells^−1^.h^−1^. When microalgae cells were heated or when tungstate was added, NR activity achieved 0.18 and 0.20 *µ*mol NO_2_^−^ 10^6^ cells^−1^.h^−1^, respectively (Figure 2(a)).

Then, different sodium tungstate concentrations were tested. Indeed, at 5 mM of sodium tungstate, NR activity started to be affected. Addition of 5 mM, 10 mM, or 15 mM of sodium tungstate caused significant decrease of NR activity reaching a critical level 0.43 *µ*mol NO_2_^−^ 10^6^ cells^−1^.h^−1^ by adding 15 mM of tungstate (Figure 2(b)).

### 3.3. Lipid Accumulation

**Figure 3:**
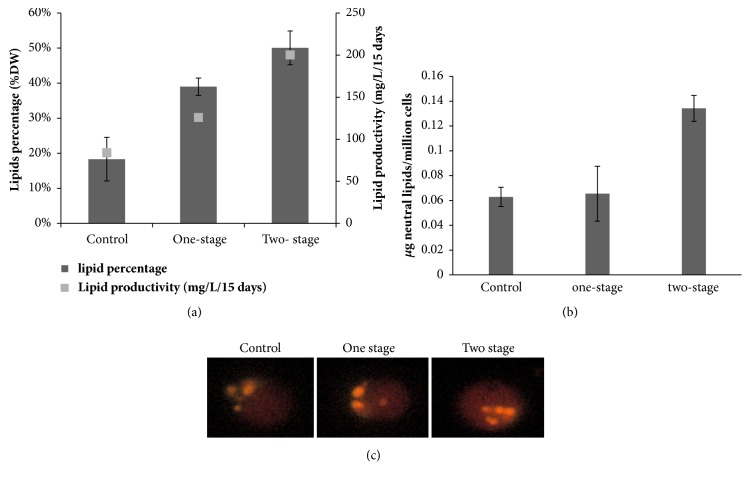
Effect of tungstate on total and neutral lipid accumulation in* D. tertiolecta*. (a) Lipid content (histogram) and lipid productivity per cycle (squares). (b) Neutral lipids droplets accumulation in* D. tertiolecta* stained with Nile red dye. (c) Neutral lipid analysis by spectrofluorimetry expressed as *µ*g of neutral lipids/millions of cells. All cultures were cultivated in Guillard F/2 medium. Tungstate (10 mM) was added to the cultures at the beginning of culture one-stage treatment or at 7th day ‘two-stage treatment'. Data are mean ± standard deviation.

Total lipids on the 15th day were determined by gravimetric analysis in order to investigate the effect of tungstate treatment on lipids accumulation. Total lipids yield showed a significant increase with the two-stage culture strategy. In fact, lipids content of* D. tertiolecta* was 18% in normal conditions and increased by tungstate treatment and reached 39% and 50% in culture on single-stage and two-stage of culture, respectively (Figure 3(a)). This improvement of lipids in one-stage culture strategy was accompanied by a decrease in growth. In terms of lipid productivity, it was ameliorated by one-stage treatment from 84 (control) to 126 mg/L/15 days, and lipid productivity was enhanced by two-stage culture reaching 200.2 mg/L/15 days.

### 3.4. Neutral Lipids

Since neutral lipids are the real feedstock for biodiesel production, a real time monitoring of triacylglycerols (TAG) was carried out using fluorescent microscopy under tungstate treatment of* D. tertiolecta*, by observing the accumulation of neutral lipid bodies. After staining cells with Nile red fluorescence dye we show in Figure 3(b) that nitrogen stress caused an accumulation of TAG. Pictures show the presence of multiple TAG containing particles in microalgae under nitrogen limited conditions for both treatments.

Next, neutral lipids were quantified by spectrophotometric method. Cells grown under tungstate treatment in two-stage conditions exhibited an enhancement of neutral lipids fluorescence from 65.46 .10^−3^ to 134.23 .10^−3^ *µ*g/million 2.14 fold more than control cells (Figure 3(c)).

### 3.5. FAME Analysis and Physicochemical Properties of the Biodiesel

Fatty Acids Methyl Esters (FAME) profile influences directly the biodiesel quality. The effect of tungstate treatment of FAME profile was analyzed. After basic transesterification of total lipids extracted from microalgae cultured in normal conditions and those treated by tungsten, resultant Fatty Acids Methyl Esters (FAMEs) were analyzed by GC-MS and the profile of corresponding fatty acid was presented in Table 1.

Then physical properties of biodiesel composed from cetane number, kinematic viscosity, kinematic viscosity, density and higher heating value were calculated in Table 2. We showed that higher heating value was increased by tungstate treatment while cetane number remained steady.

### 3.6. Chlorophyll Analysis

**Figure 4:**
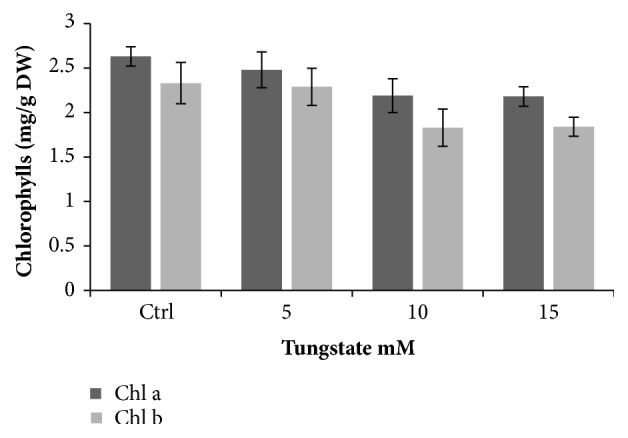
Effect of tungstate dose on chlorophyll a and b of* D. tertiolecta *at 15 days of culture on F/2. Tungstate was added at 7th day of culture. Data are mean ± standard deviation.

To get an idea on photosynthetic efficiency which is strongly related to nitrogen assimilation, chlorophyll was analyzed. Chlorophyll a and b remained steady when adding 5 mM of tungstate and decreased slightly by adding 10 mM and 15 mM tungstate, from 2.63 to 2.18 for the chlorophyll a and from 2.33 to 1.84 for the chlorophyll b, which means a loss of 19% of total chlorophyll (a and b) (Figure 4).

## 4. Discussion

We showed that the addition of tungstate to the microalgal cultures led to a decrease of NR activity in* Dunaliella tertiolecta *(Figure 2). Addition of tungsten which is the antagonist of molybdenum to the culture medium inhibits NR and subsequently inhibits the growth of microalgae by nitrogen depletion (Figure 1) [12, 15, 21]. Indeed, nitrogen constitutes a major fraction of the biomass, being essential component in nucleic acids, amino acids, chlorophyll et cetera. Using two-stage culture, tungstate did not significantly affect growth rate since cells were close to their maximum of growth. Several studies were conducted with success using the two-stage strategy in order to enhance lipid productivity while kipping the same growth rate [5, 11, 23, 24].

Ostensibly, nitrogen limitation is the most critical nutrient affecting lipid accumulation in microalgae and synthesis and sequestration of triglycerides into cytosolic lipid bodies and this may be one of protective mechanisms by which microalgae cope with nitrogen stress condition [25]. Interestingly, nitrogen stress by sodium tungstate treatment was accompanied by lipid metabolism change leading to the accumulation of fatty acids in* P. tricornutum* [26]. For example, pyruvate dehydrogenase E1 component, which catalyzes the first step of pyruvate dehydrogenase complex, showed an increase of expression under nitrogen deprivation, and this accelerates the conversion of pyruvate to acetyl-CoA. Acetyl-CoA may be used in the citric acid cycle to produce fatty acids in [26]. This suggestion is confirmed by showing that citrate synthase was upregulated under the same stress [27]. It has been also reported that Phosphomannomutase, Transaldolase, and Glyceraldehyde 3-phosphate dehydrogenases were regulate towards the accumulation of fatty acids under nitrogen deprivation [26]. The expression level of enzymes involved in the biosynthesis of triglycerides was studied also. Indeed, expressions of Acetyl-CoA carboxylase and diacylglycerol acyltransferase catalyzing the first and the last step in triglycerides biosynthesis were studied in* Chlorella pyrenoidosa *[28] and transcript levels of genes coding for these enzymes were found to increase dramatically under nitrogen starvation and this was accompanied by an increment of triglycerides [28].

We showed also the reduction of chlorophyll under tungstate treatment (Figure 4). This was demonstrated by others studies [29, 30]. Effectively, reduction of 28% of chlorophyll a was observed when* D. tertiolecta* cells were nitrogen starved [30]. Consequently, photosynthetic efficiency was reduced upon nitrogen limitation in many species including* D. tertiolecta* [27, 31]. This could be explained by the nitrogenous composition of chlorophyll and by the fact that many proteins involved in the synthesis of chlorophyll and its precursors, such as geranylgeranyl reductase, were downregulated under nitrogen starvation [27].

FAME profile was also studied and showed a slight increase in polyunsaturation and elongation (Table 1). Interestingly, enoyl-acyl carrier protein reductase, one of the major enzymes involved in lipid metabolism, was regulated under nitrogen stress in* P. tricornutum *[26]. This enzyme catalyzes the reduction of instaurations and elongation cycle of fatty acids. On the other hand, polyunsaturations are generally high in microalgae according to European Standard EN 14214 for biodiesel production. Some solutions were proposed such as partial hydrogenation [32].

Physical proprieties of produced biodiesel were predicted from FAME composition (Table 2). Viscosity is an important fuel parameter because it affects the atomization quality [16]. ASTM D-6751 standard requires 1.9 to 6 mm^2^/s as the limits of Kinematic viscosity at 40°C. Both treated and nontreated cultures meet standard requirement. Another important property is the higher heating value which is the amount of heat produced by the complete combustion. This value was improved from 30.3 to 34.5 (MJ/kg) by adding sodium tungstate, approaching thereby the petroleum diesel (46 MJ/kg).

In medicine, tungsten is used as antidiabetic drug. Indeed, it is an effective antidiabetic agent with a minimal side effect [33, 34]. Naturally, it is present in some agricultural fertilizers in proportions of 100 mg/Kg soil dry [35], but at high concentrations, tungsten has an impact on microbial population of soil by increasing fungi and reducing bacteria [36]. No observed effect concentration (NOEC) of ≥ 10g/kg dry soil was reported on earthworm (*Eisenia fetida*) survival and reproduction [36, 37]. For terrestrial plants, toxicity of sodium tungstate is variable among species and NOEC was 10000, 111 and 37 mg/Kg dry soil for respectively lettuce, radish, and oat [37].

The concentrations used in our study (5 mM or 10 mM) were not high as compared as toxic dose for environment; furthermore, tungsten is incorporated in microalgae cells and used in their metabolism, which mitigates tungsten in culture medium from cycle to another.

Two-phase nitrogen stress culture requires centrifugation of biomass and culture in a new nitrogen-free medium which is expensive and laborious. The addition of tungstate at specific stage of culture is considered as an interesting solution regarding its low-cost and ease of the process. Hence, we consider that the use of tungstate according our method is relevant as the concentrations used are low and the price of sodium tungstate is affordable.

## 5. Conclusions

Sodium tungstate had its effect on inhibition of Nitrate reductase in* D. tertiolecta* cells and induced the biosynthesis of neutral lipids while keeping high growth rate using two-stage culture strategy. This was accompanied by some changes in cell metabolism. This finding has its importance for microalgae-based integrated biodiesel system by improving both lipid productivity of* Dunaliella Tertiolecta *and quality for biodiesel production.

## Figures and Tables

**Table 1:** Fatty acid methyl ester (FAMEs) profile of *D. tertiolecta *analyzed by GC-MS at the 15th day. Sodium tungstate (10 mM) was added to the cultures at the beginning of culture one-stage treatment. PUFA: polyunsaturated fatty acids; SFA: saturated fatty acids; UFA: unsaturated fatty acids.

		**Control**	**Tungstate**
**C14:0**	myristic acid methyl ester	0.93	0.85
**C16:4**	hexadecatetraenoic acid methyl ester	7.65	9.4
**C16:3**	hexadecatrienoic acid methyl ester	2.2	2.41
**C16:2**	hexadecadienoic acid methyl ester	0	0.4
**C16:0**	palmitic acid	26.81	26.03
**C17:3**	heptadeca-5,8,11-trienoic acid methyl ester	0.98	0.6
**C18:3**	Linolenic methyl ester	24.84	30.11
**C18:2**	Linoleic acid methyl ester	5.83	8.56
**C18:0**	Stearic acid methyl ester	7.32	5.86
**C20:4**	eicosatetraenoic acid methyl ester	0	2.64
**C20:3**	eicosatrienoic acid methyl ester	0	0.13
**C20:0**	arachidic acid methyl ester	0.16	0.26
**PUFA≥4**		7.65	12.04
**SFA**		35.22	33
**UFA**		41.5	54.25

**Table 2:** Physicochemical properties of the biodiesel using empirical equations in treated and nontreated culture by sodium tungstate (10 mM).

	**Cetane number**	**Kinematic viscosity, 40**°**C (mm** ^**2**^ **/s)**	**Density, 20**°**C (g/cm** ^**3**^ **)**	**Higher heating value (MJ/kg)**
**control**	33.85	2.85	0.68	30.37
**Tungstate**	33.43	3.14	0.77	34.54

## Data Availability

The data used to support the findings of this study are included within the article.
